# The effects of changing climate on faunal depth distributions determine winners and losers

**DOI:** 10.1111/gcb.12680

**Published:** 2014-08-01

**Authors:** Alastair Brown, Sven Thatje

**Affiliations:** Ocean and Earth Science, University of SouthamptonEuropean Way, Southampton SO14 3ZH, UK

**Keywords:** bathymetric, climate change, fundamental ecological niche, hydrostatic pressure, oxygen, physiology, range shift, temperature

## Abstract

Changing climate is predicted to impact all depths of the global oceans, yet projections of range shifts in marine faunal distributions in response to changing climate seldom evaluate potential shifts in depth distribution. Marine ectotherms' thermal tolerance is limited by their ability to maintain aerobic metabolism (oxygen- and capacity-limited tolerance), and is functionally associated with their hypoxia tolerance. Shallow-water (<200 m depth) marine invertebrates and fishes demonstrate limited tolerance of increasing hydrostatic pressure (pressure exerted by the overlying mass of water), and hyperbaric (increased pressure) tolerance is proposed to depend on the ability to maintain aerobic metabolism, too. Here, we report significant correlation between the hypoxia thresholds and the hyperbaric thresholds of taxonomic groups of shallow-water fauna, suggesting that pressure tolerance is indeed oxygen limited. Consequently, it appears that the combined effects of temperature, pressure and oxygen concentration constrain the fundamental ecological niches (FENs) of marine invertebrates and fishes. Including depth in a conceptual model of oxygen- and capacity-limited FENs' responses to ocean warming and deoxygenation confirms previous predictions made based solely on consideration of the latitudinal effects of ocean warming (e.g. Cheung *et al*., 2009), that polar taxa are most vulnerable to the effects of climate change, with Arctic fauna experiencing the greatest FEN contraction. In contrast, the inclusion of depth in the conceptual model reveals for the first time that temperate fauna as well as tropical fauna may experience substantial FEN expansion with ocean warming and deoxygenation, rather than FEN maintenance or contraction suggested by solely considering latitudinal range shifts.

## Introduction

Global climate change is warming the oceans (Collins *et al*., 2013). Depending on the emission scenario, projections of oceanic warming under the moderate greenhouse gas growth scenario predict an increase in global average sea surface temperature ranging from about 1 °C (RCP2.6) to more than 3 °C (RCP8.5) for the period 2081–2100, relative to the period 1986–2005 (Collins *et al*., 2013). Increasing temperature will affect taxa on the continental shelf and upper continental slope first, but the whole ocean will eventually warm up reasonably uniformly by the amount of the surface increase (Li *et al*., 2013), affecting even abyssal organisms. Marine ectotherms tend to fully occupy their thermal niches (Sunday *et al*., 2012), and it is well recognised that organisms' latitudinal ranges are responding rapidly to geographical shifts in their fundamental ecological niches (FENs; defined physiologically by the environmental variables where a species can survive; see Barve *et al*., 2011; Soberón, 2014) forced by changing climate (Pinksy *et al*., 2013). However, changes in the depth of organisms' FENs remain little considered in projections, despite clear supporting evidence for responses in shallow-water (<200 m depth) (e.g. Perry *et al*., 2005; Dulvy *et al*., 2008; Nye *et al*., 2009; Pinksy *et al*., 2013) and deep-sea (>200 m depth) taxa (e.g. Yasuhara *et al*., 2009).

Extensive experimental assessment of thermal effects on shallow-water marine organisms suggests that thermal tolerance relates directly to an organism's ability to maintain aerobic metabolism (Pörtner, 2010). Beyond the range of optimal temperature conditions, biological membrane function is significantly reduced by changes in membrane fluidity, and the efficiency of protein transcription, translation and replication is reduced by protein denaturation (Pörtner, 2010). The resulting increase in effort required for regulating and maintaining internal conditions (homeostasis) demands increased mitochondrial activity (Pörtner, 2010), for example to support elevated synthesis of protein chaperones to counteract interruption of protein structure (Pörtner, 2010). But, the increase in oxygen supply delivered by elevated ventilation and circulation does not directly match the increase in mitochondrial oxygen demand (Frederich & Pörtner, 2000; Pörtner, 2010). Consequently, although basic metabolic processes may be maintained beyond optimal conditions, processes not essential to basic life support, such as growth, reproduction, feeding, and voluntary movement, are reduced (Pörtner, 2010). At a population level, this may lead to significant demographic impacts over time; reductions in growth and subsequently in reproductive output may affect species' survival (Pörtner, 2010). For the individual, passing the critical threshold, where mitochondrial oxygen demand exceeds the respiratory capacity of the animal, results in a mismatch of oxygen supply and demand and anaerobic respiration ensues, ultimately leading to death (Pörtner, 2010). This model is commonly known as the oxygen- and capacity-limitation hypothesis.

Available evidence supports a functional association between thermal and hypoxic tolerance (Anttila *et al*., 2013); for example, hyperoxia has been shown to increase the critical thermal limit of the Antarctic marine bivalve *Laternula elliptica* (Pörtner *et al*., 2006). Consequently, hypoxia is hypothesised to exacerbate oxygen limitation of thermal niches, narrowing thermal performance windows and reducing thermal ranges (Pörtner, 2010). Ocean warming and increasing stratification are causing declines in oxygen concentration in the ocean interior, which may be compounded by eutrophication (Keeling *et al*., 2010). Oxygen concentration in the tropical Atlantic and Pacific has declined by 0.9–3.4 *μ*mol O_2_ kg^−1^ per decade in the 300–700 m layer, with a vertical expansion of the hypoxia zone as the depth of the 60 *μ*mol O_2_ kg^−1^ hypoxic horizon shoaled from 245 to 170 m in the eastern Pacific (Stramma *et al*., 2008). In the North Pacific, the best resolved region, declines of 12 *μ*mol O_2_ kg^−1^ per decade occurred between 1987 and 2006 at 1300 m depth off British Columbia (Whitney *et al*., 2007). Similar magnitude declines occurred from 1984 to 2004 off the coast of Southern California, associated with a shoaling of the hypoxic boundary by up to 100 m (Bograd *et al*., 2008). Models predict declines of between 1% and 7% in average ocean oxygen concentration by 2100, which predominantly affect waters of the upper continental slope and shallower (Keeling *et al*., 2010). Decreasing oxygenation of deeper waters is likely to reduce tolerance of the lower temperatures that prevail with increasing depth, decreasing the depth of FENs. However, the bathymetric (depth related) limit of FENs may not be determined by oxygen concentration and temperature alone.

The extant deep-sea fauna are understood to derive predominantly from colonisations and radiations by shallow-water organisms, and consequently widespread patterns of bathymetric zonation on continental margins have been interpreted as evidence of a physiological bottleneck, imposed by the effects of high pressure and low temperature (Brown & Thatje, 2011, 2014). Low temperature and high hydrostatic pressure are, therefore, perceived as key factors that physiologically limit submergence of shallow-water taxa (Brown & Thatje, 2014).

Technical challenges have restricted experimental assessments of physiology under sustained pressure, and there is little evidence of the mechanisms constraining pressure tolerance. However, limited observations of respiratory and cardiac responses to pressure change appear to support the application of the oxygen- and capacity-limitation hypothesis to hydrostatic pressure tolerance (Mickel & Childress, 1982a,b; Airriess & Childress, 1994; Thatje & Robinson, 2011). There are also consistent indications that voluntary movement and feeding are affected by hyperbaric conditions beyond optimum (Thatje *et al*., 2010; Oliphant *et al*., 2011; Thatje & Robinson, 2011). If oxygen- and capacity-limitation constrains tolerance of increased hydrostatic pressure, *a priori* assumptions suggest that any variation in hyperbaric tolerance among taxonomic groups would be significantly correlated with variation in hypoxia tolerance among taxonomic groups. We test this hypothesis by comparing hypoxic thresholds of different benthic marine groups, identified using a data set compiled by Vaquer-Sunyer & Duarte (2008), with hyperbaric thresholds of different benthic marine groups, identified by pooling data from existing hyperbaric studies. Whilst it is not possible to attribute correlation in hypoxic and hyperbaric thresholds to a specific mechanism, this can offer support for the existing hypothesis of oxygen- and capacity-limited hyperbaric tolerance. Subsequently, we construct a conceptual model to examine shifts in hypothetical oxygen- and capacity-limited FENs of shallow-water taxa in different thermal zones in response to predicted changes in ocean temperature and oxygenation under stabilising emissions scenarios.

## Materials and methods

### Data collection

We searched the literature using the keywords ‘hyperbaric’, ‘pressure’, ‘shallow-water’, and ‘marine’ and their combinations to guide the search. Published reports of shallow-water marine organisms' responses to hydrostatic pressure were then examined to identify the most sensitive measure permitting comparison across the broadest range of phyla (following groupings in Vaquer-Sunyer & Duarte, 2008 to facilitate direct comparison of taxonomic patterns in pressure thresholds and oxygen concentrations thresholds; minimum *n* = 3). Experimental assessments examining 1 h lethal pressure thresholds (statistically derived pressure at which 50% of test animals die; LP_50_) provided data for the greatest number of species of marine organisms across the widest range of taxonomic groups. Although multiple values of LP_50_ exist for several species, a single representative value for each species was required to avoid biasing the pressure threshold of any taxonomic group towards an individual species. To eliminate the potential effect of temperature stress the LP_50_ derived at the temperature most similar to maintenance or sampling temperature was selected as the representative value where studies examined thresholds at multiple temperatures. A pattern of decreasing pressure tolerance with advancing ontogeny has recently been highlighted (Mestre *et al*., 2013), therefore the LP_50_ of the most ontogenetically advanced stage was adopted as the representative value for a species where multiple life-history stages were examined. Although patterns in the hyperbaric tolerance of pelagic fauna of different taxonomic groups are expected to be similar to those of benthic and demersal fauna, it is anticipated that absolute tolerances may differ as a result of contrasting adaptations in differing habits, e.g. diel vertical migration in pelagic taxa. Insufficient data were available to represent pelagic fauna of different taxonomic groups independently from benthic and demersal fauna, and consequently pelagic taxa were excluded to reduce potential confounding by contrasting adaptations to benthic and demersal fauna.

Data for sublethal oxygen concentration thresholds of shallow-water benthic and demersal marine organisms (oxygen concentration at or below which 50% of test animals exhibit sublethal responses, or at or below which there is a significant effect on the response measure; SLC_50_) were identified from Vaquer-Sunyer & Duarte (2008). Sublethal thresholds were selected as the most sensitive measure permitting comparison across the broadest range of taxonomic groups. Data were treated as described for lethal hydrostatic pressure threshold data. Selection of data for the most advanced life-history stage was again appropriate, since hypoxia typically impacts larger animals first (Clark *et al*., 2013). Where multiple values for SLC_50_ remained, the lowest sublethal oxygen concentration value was selected as representing the most acclimatised or adapted population of the species.

### Statistical analysis

LP_50_ and SLC_50_ data (see Tables S1 and S2 in Supporting Information) were square-root transformed to achieve normality and equal variance (Shapiro–Wilk and Levene tests respectively; *P* > 0.05). anova was used to test for differences in thresholds among taxonomic groups, and the Holm–Sidak post hoc test was used to determine significant differences between mean threshold values among taxa (*α* = 0.05). Covariance in mean lethal pressure thresholds and sublethal oxygen concentration thresholds was examined by testing their correlation (Pearson product-moment correlation).

### Modelling geographical shifts in hypothetical oxygen- and capacity-limited FENs in response to ocean warming and deoxygenation

Projected increases in temperature (°C) and decreases in oxygen concentration (*μ*mol kg^−1^) by 2100 under stabilising emissions scenarios were identified from Collins *et al*., 2013 (RCP4.5) and Matear & Hirst, 2003 (IS92a). Marine taxon range shifts are tightly coupled to shifts in thermal envelope (Pinksy *et al*., 2013). Therefore, the magnitude of anticipated climate warming effects on thermally constrained latitudinal ranges of hypothetical oxygen- and capacity-limited FENs was estimated based on the best supported mean temperature effects on leading-edge expansions and trailing-edge contractions of distributions (30.6 ± 5.2 km dec^−1^) (Poloczanska *et al*., 2013), as temperatures have increased (0.07 °C dec^−1^) (Burrows *et al*., 2011). Mean rates were used because the disparity in leading-edge expansions and trailing-edge contractions likely derives predominantly from geographical variation in climate velocity (Pinksy *et al*., 2013; Burrows *et al*., 2014). This yielded leading-edge expansion and trailing-edge contraction rates of ∽440 km °C^−1^; latitudinal shifts were rounded to the nearest 10 km. The magnitude of anticipated thermal effects on hyperbaric limits to hypothetical oxygen- and capacity-limited FENs was estimated based on average experimentally determined temperature effects on the critical hyperbaric thresholds of postlarval shallow-water tropical (+90 m depth °C^−1^; *n* = 3; three crustaceans) (Menzies & George, 1972), temperate (+50 m depth °C^−1^; *n* = 2; two crustaceans) (Thatje *et al*., 2010; Oliphant *et al*., 2011) and polar (−330 m depth °C^−1^; *n* = 2; one mollusc and one crustacean) (George, 1979; Smith & Thatje, 2012) taxa; bathymetric shifts were rounded to the nearest 10 m. Insufficient data were available to estimate the magnitude of effects of ocean deoxygenation on FENs, therefore arbitrary effects of oxygen changes (10 km latitude *μ*mol^−1^ O_2_, and 1 m depth *μ*mol^−1^ O_2_) were modelled to provide an indication of relative impacts on FENs in different thermal zones. Hypothetical FENs initially span 10° (polar) or 20° (temperate and tropical) latitude and are 300 m deep and were selected to represent hypothetical oxygen- and capacity-limited species with distributions in the areas of the global oceans most severely impacted by changing climate. Shifts in cross-sectional area of FENs in response to projected ocean warming were calculated, but no calculation was made for responses to projected ocean deoxygenation or combined ocean warming and deoxygenation, since deoxygenation impacts were assigned arbitrarily.

## Results

### Hyperbaric thresholds and hypoxic thresholds of different taxonomic groups

**Fig 1:**
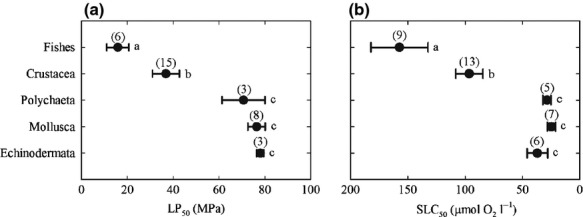
Distributions of (a) hyperbaric and (b) hypoxic tolerance thresholds among shallow-water marine benthic invertebrates and demersal fishes. Hyperbaric LP_50_ and hypoxic SLC_50_ tolerance thresholds identified by analysis of pooled data from published studies. Error bars represent standard error; numbers in brackets represent the number of species used to calculate the mean value of each taxonomic group. Letters indicate significant differences.

Lethal pressure thresholds and sublethal oxygen concentration thresholds are significantly different among marine taxa (*F*_4,30_ = 15.033, *P* < 0.001, and *F*_4,35_ = 12.321, *P* < 0.001 respectively), and show strikingly similar patterns of variation (Fig.1a and b). Fishes are significantly less tolerant of increased pressure or decreased oxygen concentration than all other taxonomic groups. Crustaceans are significantly more tolerant of increased pressure or decreased oxygen concentration than fishes, but significantly less tolerant of these challenges than polychaetes, molluscs and echinoderms. There are no significant differences between the tolerances of polychaetes, molluscs or echinoderms to increased pressure or decreased oxygen concentration. Mean lethal pressure thresholds and sublethal oxygen thresholds are significantly inversely correlated (Pearson's *r* = −0.983, df = 3, *P* = 0.003; *r*^2^ = 0.966).

### Projected geographical shifts in hypothetical oxygen- and capacity-limited FENs in response to ocean warming and deoxygenation

**Fig 2:**
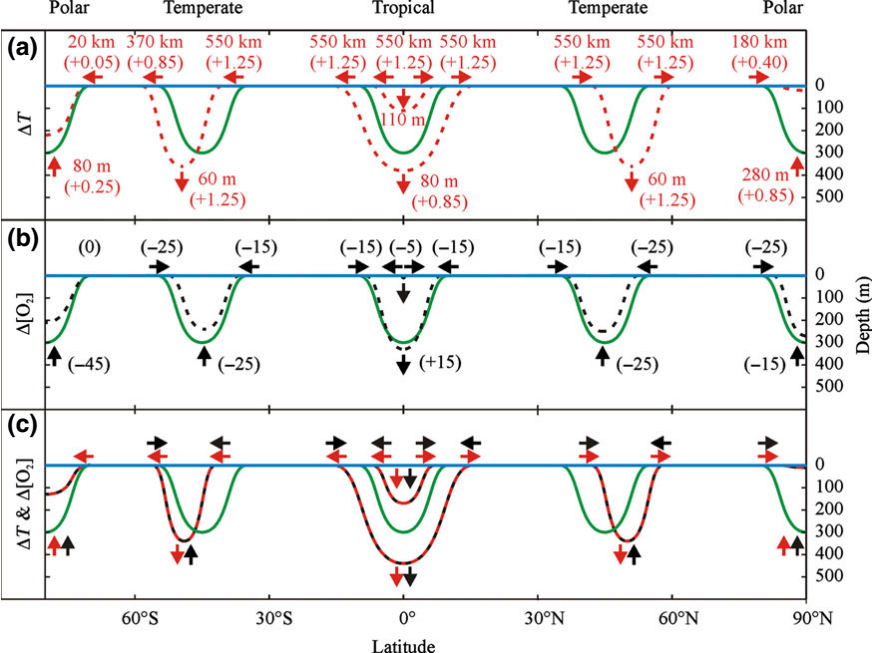
Projected shifts in fundamental ecological niches (FENs) in response to (a) increasing ocean temperature (b) decreasing ocean oxygenation, and (c) increasing ocean temperature and decreasing ocean oxygenation, assuming oxygen- and capacity- limitation of thermal, hyperbaric and hypoxic tolerance. FENs initially span 10° (polar) or 20° (temperate or tropical) latitude and are 300 m deep. Arrows indicate projected effects of increasing temperature (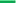) and decreasing oxygenation (─) on hypothetical FENs (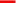) in tropical, temperate, and polar zones. The magnitude of anticipated thermal effects on latitudinal and bathymetric limits to FENs is indicated alongside arrows, with dashed lines indicating resulting FENs. Projected increases in temperature (°C) and decreases in oxygen concentration (*μ*mol O_2_ kg^−1^) by 2100 are indicated in brackets. Data for the effects of decreasing oxygen concentration on thermal or hyperbaric limits are not available, and consequently no magnitude of deoxygenation effect is indicated. However, dashed lines are used to provide an indication of relative impacts of ocean deoxygenation on FENs.

Latitudinal limits to all FENs shifted poleward under projected ocean warming (Fig.2a). Both the upper and lower bathymetric limits of the tropical FEN were depressed (Fig.2a). The lower bathymetric limit of the temperate FENs was also depressed, but the lower limit of the polar FENs shoaled (Fig.2a). Tropical and temperate FENs increased in cross-section in response to projected ocean warming, whereas the polar FENs decreased in cross-section (Table1).

**Table 1:** Impact of predicted ocean warming to the year 2100 on the cross-sectional area of hypothetical oxygen- and capacity-limited fundamental ecological niches (FENs) from the year 2000

FEN	Cross-sectional area			
	2000 (km^2^)	2100 (km^2^)	Change (km^2^)	% Change
Polar northern hemisphere	165	9	−156	−94
Temperate northern hemisphere	330	396	+66	+20
Tropical	330	597	+267	+81
Temperate southern hemisphere	330	364	+34	+10
Polar southern hemisphere	165	119	−46	−28

Areas were calculated from Fig.2.

Latitudinal limits to all FENs were constricted under projected ocean deoxygenation (Fig.2b). Both the upper and lower bathymetric limits of the tropical FEN were depressed (Fig.2b). In contrast, the lower bathymetric limits of temperate and polar FENs shoaled (Fig.2b). The effects of ocean deoxygenation compounded ocean warming induced shifts in trailing-edges of FENs, but moderated shifts in leading-edges (Fig.2c). Whilst shifts in bathymetric limits of tropical and polar FENs induced by ocean warming were compounded by ocean deoxygenation, ocean deoxygenation moderated shifts in the bathymetric limits of temperate FENs caused by ocean warming (Fig.2c).

## Discussion

Caution is required in interpreting the significant correlation between hypoxic and hyperbaric thresholds evidenced here, since available data are limited and the taxonomic sampling is not paired, and since correlation does not necessarily indicate causation. However, the correlation supports the application of the oxygen- and capacity-limitation hypothesis, originally conceived to explain thermal tolerance (Pörtner, 2010), to hydrostatic pressure tolerance. It is, perhaps, unsurprising that both thermal and hyperbaric tolerance may be limited by the same mechanism; the effects of high pressure on biological membranes and proteins are similar to the effects of low temperature, and low temperature and high hydrostatic pressure both require increases in homeostatic effort (Brown & Thatje, 2014). Oxygen limitation of thermal tolerance depends proximately on capacity-limitation of ventilation and circulation (Pörtner, 2002). Progressive failure of the thermally sensitive membrane transporter Na^+^ K^+^ ATPase contributes to thermal limitation of cardiac capacity (Stillman, 2002); Na^+^ K^+^ ATPase is critical to maintaining membrane potential needed to generate action potentials in excitable cells such as those regulating cardiac function (see Lodish *et al*., 2003). Progressive failure of Na^+^ K^+^ ATPase likely results from thermal effects on membrane fluidity, which stimulate conformational changes in membrane-bound proteins, progressively impacting protein function (Tillman & Cascio, 2003). Lipid bilayers of biological membranes are one of the most pressure sensitive cellular structures (Somero, 1992). Pressure increase reduces the fluidity of biological membranes; a pressure increase of 10 MPa (≈1000 m water depth) is equivalent to a decrease in temperature of ∽1.3–2.1 °C, depending on membrane composition (Somero, 1992). Pressure effects on membrane fluidity stimulate conformational changes in membrane-bound proteins too (Tillman & Cascio, 2003), and Na^+^ K^+^ ATPase also fails progressively under hyperbaric conditions (Gibbs & Somero, 1989), potentially causing observed hyperbaric decreases in excitatory junction potential amplitude at the neuromuscular junction by impeding neurotransmitter release (Campenot, 1975), and thus hampering cardiac capacity. Critical pressure effects on Na^+^ K^+^ ATPase are supported by enzymatic functional pressure-adaptation in deep-sea organisms (Gibbs & Somero, 1989). Mechanisms of thermal and hyperbaric limitation of cardiac capacity are therefore expected to be similar. Increased homeostatic effort through, for example, elevated protein chaperoning, may, to some extent, mediate the effects of hydrostatic pressure (Cottin *et al*., 2012). However, at low temperature, increased mitochondrial oxygen demand required for increased homeostatic effort is not matched by the increase in respiratory capacity delivered through elevated ventilation and circulation (Frederich & Pörtner, 2000); ventilation and circulation will also fail to deliver required oxygen levels under high pressure conditions, and survival will, therefore, be time-limited beyond the critical threshold, where anaerobic mitochondrial respiration ensues. Consequently, it is the combined physiological effects of temperature, hydrostatic pressure, and oxygen concentration that constrain the depth ranges of marine ectotherms, in a matrix of oxygen- and capacity-limited tolerance (adapted from Pörtner, 2010). Elevated carbon dioxide concentration may also integrate into a matrix of oxygen- and capacity-limited tolerance too (Pörtner, 2008); increasing carbon dioxide concentration can narrow thermal tolerance windows (e.g. Walther *et al*., 2009). Despite increasing recognition of the potential impact of changing carbon dioxide concentration (e.g. Brewer & Peltzer, 2009), zonal mean sections (latitude vs. depth) identifying carbon dioxide concentration changes projected by global ocean models under stabilising emissions scenarios are not currently available. Incorporating effects of changing carbon dioxide concentration on FENs into our conceptual model was, therefore, not possible.

Distinct adaptations of organisms in tropical, temperate and polar regions suggest that their FENs will be affected differently by ocean warming (Somero, 2012) (Fig.2a). Temperature increases will shift the upper bathymetric boundaries of the tropical FEN downwards, but increasing temperature will also mediate the effects of high pressure on tropical fauna, increasing hyperbaric tolerance and shifting the lower bathymetric limit downwards, too. Consequently, ocean warming will expand the latitudinal range of the tropical FEN substantially (Table1). The poleward shift in temperate FENs will also be accompanied by depression of the lower bathymetric limits (Fig.2a). For example, the pressure tolerance of the temperate shallow-water hermit crab *Pagurus cuanensis* increases from the equivalent of 200 m water depth to the equivalent of 500 m water depth with a 5 °C temperature increase (Thatje *et al*., 2010). Based on this, the 1.0–1.5 °C temperature increase predicted for 200–400 m water depth in the northern temperate zone by 2100 (Collins *et al*., 2013) will shift the lower bathymetric limit of the FEN downwards by 60–90 m water depth. Although this increase appears modest, depths between 200 and 300 m constitute ∽1% of the total surface area of the global ocean (Brown & Thatje, 2014). Depths from the sea surface to 200 m constitute ∽6% of the total surface area of the global ocean (Brown & Thatje, 2014). Clearly, a 60–90 m downward shift in depth limit represents a substantial expansion in FEN. Such an expansion is sufficient to offset latitudinal contraction of the southern temperate FEN in our model, resulting in an overall increase in both temperate FENs (Table1). Whilst the hyperbaric tolerance of tropical and temperate taxa is increased by increasing temperature (Brown & Thatje, 2014), the hyperbaric tolerance of cold-adapted polar taxa appears reduced (Smith & Thatje, 2012). The consequent shoaling of the lower bathymetric limits of polar FENs, together with a poleward shift in the latitude of upper thermal limits, mean that polar FENs will contract with increasing temperature (Table1).

Tropical, temperate, and polar FENs will all contract latitudinally and bathymetrically as a consequence of decreasing ocean oxygenation (Fig.2b). But the magnitude of the resulting FEN contraction remains entirely uncertain in the absence of experimental studies assessing the impact of deoxygenation on marine organisms' thermal and hyperbaric limits, and will depend on the degree of deoxygenation, which varies regionally. Nevertheless, the hypothesised deoxygenation-related FEN contraction will compound the contractive effect of ocean warming on the FENs of polar taxa, and will mediate the expansion of temperate and tropical FENs caused by rising temperature. Consequently, it appears clear that FENs in tropical, temperate and polar zones will be affected differently by components of climate change (Fig.2c). Including depth in the model confirms previous predictions that polar taxa are most vulnerable to the effects of climate change, made based solely on consideration of the latitudinal effects of ocean warming (e.g. Cheung *et al*., 2009), with Arctic fauna experiencing the greatest habitat contraction. In contrast, the inclusion of depth in the model reveals for the first time that temperate fauna as well as tropical fauna may experience substantial FEN expansion with ocean warming and deoxygenation, rather than FEN maintenance (northern hemisphere temperate and tropical) or contraction (southern hemisphere temperate) suggested by solely considering latitudinal range shifts (Fig.2).

Climate projections to 2300 predict that sea surface and subsurface temperatures will rise by between 6 and 7 °C at low latitudes and ∽10 °C at high latitudes, with the deep-sea warming by 2–5 °C (Schmittner *et al*., 2008). By the year 3000 global mean oxygen concentration will decline by 30%: shallow subsurface ocean oxygen concentration in the eastern tropical Pacific and Atlantic will decrease by more than 80%, and the eastern North Pacific margin will suffer a 40–80% oxygen reduction (Schmittner *et al*., 2008). The deep ocean will experience a decrease of more than 35% (Schmittner *et al*., 2008). Occurrences of widespread hypoxia are predicted to increase, as the volume of the total ocean that is hypoxic (defined in this case as ≤80 *μ*mol O_2_ kg^−1^) rises from 9.1% currently, to 61% around 5000 (Shaffer *et al*., 2009). Based on these projections, changing climate will continue to force shifts in FENs for millennia.

The variation in sensitivity to oxygen- and capacity-limitation among taxa, revealed here, and in sensitivity to carbon dioxide concentrations (Kroeker *et al*., 2013), suggests that responses to ocean warming, deoxygenation, and increasing carbon dioxide concentration, will differ among taxonomic groups. This is already evident from recent analysis of the global imprint of climate change on marine life, where bony fish demonstrate the greatest change in latitudinal distribution among animals (Poloczanska *et al*., 2013), and may also be expected with respect to depth distribution. Whether, and to what extent, taxa are able to shift their distribution to match the shift in their FEN depends on habitat availability (e.g. continental shelf and slope area for benthic species), suitability (e.g. primary productivity and food availability, seasonality), and accessibility (e.g. dispersal ability, current direction), amongst other factors (see Barve *et al*., 2011 and references cited therein). Light penetration may be a particularly critical limitation for bathymetric range increases in animals with vision too; adaptations in vision shift with bathymetric changes in light parameters (Warrant & Locket, 2004). Further, there is great regional variability in changing climate (e.g. Pinksy *et al*., 2013). Undoubtedly, differences in FEN shifts, and in organism's ability to respond to such shifts, will contribute to the development of no-analogue communities, with the potential for changes in ecosystem functioning (see Williams & Jackson, 2007).

Although the effects of changing climate inferred here from the matrix of thermal, hyperbaric, and hypoxic oxygen limitation do not incorporate preadaptation or adaptation to changing climate, it remains unclear whether adaptation in marine ectotherms can match rapid change in climate over time (Munday *et al*., 2013); the most warm-adapted organisms may not have capacity for any further thermal adaptation (Storch *et al*., 2014). During past extinction events, temperature, oxygen concentration and carbon dioxide concentration all experienced significant perturbations (McClain & Hardy, 2010; Hönisch *et al*., 2012; Bijima *et al*., 2013). The magnitude of future disturbance in these factors and their interaction with hydrostatic pressure effects may make a mass extirpation of life in deep waters inevitable in the long term (adapted from Jackson, 2010), but the results presented here suggest that, at least in the shorter term, there will be winners as well as losers.

## Supporting Information

Table S1 1 h lethal pressure threshold data and sources.

Table S2 Sublethal oxygen concentration threshold data and sources.
