# Supplementary material for: The effects of changing climate on faunal depth distributions determine winners and losers
Source: Glob Chang Biol. 2014 Aug 1;21(1):173–80. doi: 10.1111/gcb.12680 (PMC4310292; doi:10.1111/gcb.12680)
Supplement: Table S1 — 1 h lethal pressure threshold data and sources. [file gcb0021-0173-sd1.doc]

**Table S1.** 1 hour lethal pressure threshold (statistically derived pressure at which 50% of test animals die; LP_50_) data and sources.

| Taxon | Species | LP_50_ (MPa) | Source |
| --- | --- | --- | --- |
| **CRUSTACEA** | *Balanus* sp. | 89 | Selvakumaran *et al*., 1974 |
|  | *Carcinus maenas* | 34 | Naroska, 1968 |
|  | *Crangon crangon* | 23 | Naroska, 1968 |
|  | *Eupagurus bernhardus* | 12 | Naroska, 1968 |
|  | *Eurypanopeus* sp. | 23 | Menzies & Selvakumaran, 1974 |
|  | *Gammarus oceanicus* | 55 | Naroska, 1968 |
|  | *Idotea baltica* | 50 | Naroska, 1968 |
|  | *Jaera albifrons* | 77 | Naroska, 1968 |
|  | *Latreutes fucorum* | 20 | Menzies & Selvakumaran, 1974 |
|  | *Lepidactylus* sp | 42 | Menzies & Selvakumaran, 1974 |
|  | *Pagurus longicarpus* | 16 | Menzies & Selvakumaran, 1974 |
|  | *Petrolisthes armatus* | 19 | Menzies & Selvakumaran, 1974 |
|  | *Sesarma reticulatum* | 29 | Selvakumaran *et al*., 1974 |
|  | *Sphaeroma quadridentatum* | 40 | Menzies & Selvakumaran, 1974 |
|  | *Uca pugilator* | 23 | Avent, 1974 |
|  |  |  |  |
| **ECHINODERMATA** | *Amphioplus macilentus* | 79 | Selvakumaran *et al*., 1974 |
|  | *Asterias rubens* | 75 | Naroska, 1968 |
|  | *Psammechinus miliaris* | 80 | Naroska, 1968 |
|  |  |  |  |
| **FISHES** | *Fundulus heteroclitus* | 9 | Selvakumaran *et al*., 1974 |
|  | *Platichthys flesus* | 14 | Naroska, 1968 |
|  | *Pleuronectes platessa* | 15 | Naroska, 1968 |
|  | *Stephanolepis hispidus* | 18 | Menzies & Selvakumaran, 1974 |
|  | *Urophycis* sp. | 2 | Selvakumaran *et al*., 1974 |
|  | *Zoarces viviparus* | 37 | Naroska, 1968 |
|  |  |  |  |
| **MOLLUSCA** | *Aplysia protea* | 83 | Menzies & Selvakumaran, 1974 |
|  | *Arctica islandica* | 73 | Naroska, 1968 |
|  | *Donax variabilis* | 56 | Menzies & Selvakumaran, 1974 |
|  | *Littorina irrorata* | 94 | Menzies & Selvakumaran, 1974 |
|  | *Littorina littorea* | 75 | Naroska, 1968 |
|  | *Modiolus modiolus* | 75 | Naroska, 1968 |
|  | *Mya arenaria* | 75 | Naroska, 1968 |
|  | *Mytilus edulis* | 80 | Naroska, 1968 |
|  |  |  |  |
| **POLYCHAETA** | *Arenicola marina* | 52 | Naroska, 1968 |
|  | *Nereis diversicolor* | 78 | Naroska, 1968 |
|  | *Nereis occidentalis* | 82 | Selvakumaran *et al*., 1974 |

**References:**

Avent RM (1974) The effects of hydrostatic pressure on living aquatic organisms VIII. Behavioural and metabolic responses of *Uca pugilator* to variations in hydrostatic pressure and temperature. International Review of Hydrobiology, **59**, 219-238.

Menzies RJ, Selvakumaran M (1974) The effects of hydrostatic pressure resistance on living aquatic organisms V. Eurybiotic environmental capacity as a factor in high pressure tolerance. International Review of Hydrobiology, **59**, 199-205.

Naroska V (1968) Vergleichende Untersuchungen über den Einfluss des hydrostatischen Druckes auf Überlebensfähigkeit und Stoffwechselintensität mariner Evertebraten und Teleosteer. Kieler Meeresforchungen, **24**, 95-123.

Selvakumaran, M., Hodson, R. & Menzies, R.J. (1974).The effects of hydrostatic pressure on living aquatic organisms VII. Size and pressure effects. International Review of Hydrobiology, **59**, 213-218.
