# Supplementary material for: The effects of changing climate on faunal depth distributions determine winners and losers
Source: Glob Chang Biol. 2014 Aug 1;21(1):173–80. doi: 10.1111/gcb.12680 (PMC4310292; doi:10.1111/gcb.12680)
Supplement: Table S2 — Sublethal oxygen concentration threshold data and sources. [file gcb0021-0173-sd2.doc]

**Table S2.** Sublethal oxygen concentration threshold (oxygen concentration at or below which 50% of test animals exhibit sublethal responses; SLC_50_) data and sources, identified from Vaquer-Sunyer & Duarte 2008.

| Taxon | Species | SLC_50_ (μmol O_2_ l^-1^) | Source |
| --- | --- | --- | --- |
| **CRUSTACEA** | *Callinectes sapidus* | 64 | Pihl *et al*., 1991 |
|  | *Calocaris macandreae* | 93 | Anderson *et al*., 1991 |
|  | *Carcinus maenas* | 116 | Hill *et al*., 1991 |
|  | *Crangon crangon* | 133 | Sandberg *et al*., 1996 |
|  | *Monoporeia affinis* | 125 | Johansson, 1997 |
|  | *Munida quadrispina* | 10 | Burd & Brinkhurst, 1984 |
|  | *Penaeus aztecus* | 92 | Renaud, 1986 |
|  | *Penaeus monodon* | 99 | Seidman & Lawrence, 1985 |
|  | *Penaeus schmitti* | 160 | Rosas *et al*., 1997 |
|  | *Penaeus setiferus* | 160 | Rosas *et al*., 1997 |
|  | *Penaeus vannamei* | 61 | Seidman & Lawrence, 1985 |
|  | *Sanduria entomon* | 64 | Johansson, 1997 |
|  | *Squilla empusa* | 77 | Pihl *et al*., 1991 |
|  |  |  |  |
| **ECHINODERMATA** | *Amphiura chiaje* | 25 | Rosenberg *et al*., 1991 |
|  | *Amphiura filiformis* | 32 | Vistisen & Vismann, 1997 |
|  | *Echinocardium cordatum* | 25 | Nilsson & Rosenberg, 1994 |
|  | *Holothuria forskali* | 27 | Astall & Jones, 1991 |
|  | *Luidia clathrata* | 81 | Diehl *et al*., 1979 |
|  | *Ophiura albida* | 32 | Vistisen & Vismann, 1997 |
|  |  |  |  |
| **FISHES** | *Callionymus lyra* | 212 | Hughes & Umezawa, 1968a |
|  | *Diplodus puntazzo* | 77 | Valverde & Garcia, 2004 |
|  | *Fundulus heteroclitus* | 144 | Voyer & Hennekey, 1972 |
|  | *Gadus morhua* | 55 | Schurmann & Steffensen, 1997 |
|  | *Gadus macrocephalus* | 144 | Alderdice & Forrester, 1971 |
|  | *Hydrolagus collie* *l* | 274 | Hanson, 1967 |
|  | *Rhacochilus vacca* | 253 | Webb & Brett, 1972 |
|  | *Scyliorhinus canicula* | 140 | Hughes & Umezawa, 1968b |
|  | *Squalus suckleyi* | 212 | Lenfant & Johansen, 1966 |
|  |  |  |  |
| **MOLLUSCA** | *Abra alba* | 19 | Jorgensen, 1980 |
|  | *Cerastoderma edule* | 19 | Jorgensen, 1980 |
|  | *Hydrobia ulvae* | 19 | Jorgensen, 1980 |
|  | *Mya arenaria* | 19 | Jorgensen, 1980 |
|  | *Mysella bidentata* | 32 | Nilsson & Rosenberg, 1994 |
|  | *Octopus vulgaris* | 145 | Valverde & Garcia, 2005 |
|  | *Theora fragilis* | 41 | Tamai, 1996 |
|  |  |  |  |
| **POLYCHAETA** | *Capitella capitata* | 26 | Warren, 1977 |
|  | *Loimia medusa* | 33 | Llanso & Diaz, 1994 |
|  | *Pectinaria koreni* | 32 | Nilsson & Rosenberg, 1994 |
|  | *Scoloplos armiger* | 36 | Schottler & Grieshaber, 1988 |
|  | *Streblospio benedicti* | 16 | Llanso, 1991 |

**References:**

Alderdice DF, Forrester CR (1971) Effects of salinity, temperature, and dissolved oxygen on early development of pacific cod (*Gadus macrocephalus*). Journal of the Fisheries Research Board of Canada, **28**, 883-902.

Anderson SJ, Atkinson RJA, Taylor AC (1991) Behavioural and respiratory adaptations of the mud-burrowing shrimp *Calocaris macandreae* Bell (Thalassinidea: Crustacea) to the burrow environment. Ophelia, **34**, 143-156.

Astall CM, Jones MB (1991) Respiration and biometry in the sea cucumber *Holothuria forskali*. Journal of the Marine Biological Association of the United Kingdom, **71**, 73-81.

Burd BJ, Brinkhurst RO (1984) The distribution of the galatheid crab *Munida quadrispina* (Benedict 1902) in relation to oxygen concentrations in British Columbia fjords. Journal of Experimental Marine Biology and Ecology, **81**, 1-20.

Diehl WJ, McEdward L, Proffitt E, Rosenberg V, Lawrence JM (1979) Response of *Luidia clathrata* (Echinodermata: Asteroidea) to hypoxia. Comparative Biochemistry and Physiology Part A, **62**, 669-671.

Hanson, D. (1967). Cardiovascular dynamics and aspects of gas exchange in Chondrichthyes. PhD thesis. University of Washington, USA.

Hill AD, Taylor AC, Strang RHC (1991) Physiological and metabolic responses of the shore crab *Carcinus maenas* (L) during environmental anoxia and subsequent recovery. Journal of Experimental Marine Biology and Ecology, **150**, 31-50.

Hughes GM, Umezawa S-I (1968a) On respiration in the dragonet *Callionymus lyra* L. Journal of Experimental Biology, **49**, 565-582.

Hughes GM, Umezawa S-I (1968b) Oxygen consumption and gill water flow in the dogfish *Scyliorhinus canicula* L. Journal of Experimetnal Biology, **49**, 557-564.

Johansson B (1997) Behavioural response to gradually declining oxygen concentration by Baltic Sea macrobenthic crustaceans. Marine Biology, **129**, 71-78.

Jorgensen BB (1980) Seasonal oxygen depletion in the bottom waters of a Danish fjord and its effect on the benthic community. Oikos, **34**, 68-76.

Lenfant C, Johansen K (1966) Respiratory function in the elasmobranch *Squalus suckleyi* G. Respiration Physiology, **1**, 13-29.

Llanso RJ (1991) Tolerance of low dissolved oxygen and hydrogen sulfide by the polychaete *Streblospio benedicti* (Webster). Journal of Experimental Biology and Ecology, **153**, 165-178.

Llanso RJ, Diaz RJ (1994) Tolerance to low dissolved oxygen by the tubicolous polychaete *Loimia medusa*. Journal of the Marine Biological Association of the United Kingdom, **74**, 143-148.

Nilsson HC, Rosenberg R (1994) Hypoxic response of two marine benthic communities. Marine Ecology Progress Series, **115**, 209-217.

Pihl L, Baden SP, Diaz RJ (1991) Effects of periodic hypoxia on distribution of demersal fish and crustaceans. Marine Biology, **108**, 349-360.

Renaud ML (1986) Detecting and avoiding oxygen deficient sea water by brown shrimp, *Penaeus aztecus* (Ives), and White Shrimp *Penaeus setiferus* (Linnaeus). Journal of Experimental Marine Biology and Ecology, **98**, 283-292.

Rosas C, Sanchez A, Diazlglesia E, Brito R, Martinez E, Soto LA (1997) Critical dissolved oxygen level to *Penaeus setiferus* and *Penaeus schmitti* postlarvae (PL10-18) exposed to salinity changes. Aquaculture, **152**, 259-272.

Rosenberg R, Hellman B, Johansson B (1991) Hypoxic tolerance of marine benthic fauna. Marine Ecology Progress Series, **79**, 127-131.

Sandberg E, Tallqvist M, Bonsdorff E (1996) The effects of reduced oxygen content on predation and siphon cropping by the brown shrimp, *Crangon crangon*. Marine Ecology, **17**, 411-423.

Schottler U, Grieshaber M (1988) Adaptation of the polychaete worm *Scoloplos armiger* to hypoxic conditions. Marine Biology, **99**, 215-222.

Schurmann H, Steffensen JF (1997) Effects of temperature, hypoxia and activity on the metabolism of juvenile Atlantic cod. Journal of Fish Biology, **50**, 1166-1180.

Seidman ER, Lawrence AL (1985) Growth, feed digestibility, and proximate body composition of juvenile *Penaeus vannamei* and *Penaeus monodon* grown at different dissolved oxygen levels. Journal of the World Mariculture Society, **16**, 333-346.

Tamai K (1996) Temporal tolerance of larval Theora fragilis (Bivalvia: Semelidae) to hypoxic conditions. Fisheries Science, **62**, 996-997.

Valverde JC, Garcia BG (2004) The effects of oxygen levels on oxygen consumption, survival and ventilatory frequency of sharpsnout sea bream (*Diplodus puntazzo* Gmelin, 1789) at different conditions of temperature and fish weight. Journal of Applied Ichthyology, **20**, 488-492.

Valverde JC, Garcia BG (2005) Suitable dissolved oxygen levels for common octopus (*Octopus vulgaris* Cuvier, 1797) at different weights and temperatures: analysis of respiratory behaviour. Aquaculture, **244**, 303-314.

Vistisen B, Vismann B (1997) Tolerance to low oxygen and sulfide in *Amphiura filiformis* and *Ophiura albida* (Echinodermata: Ophiuroidea). Marine Biology, **128**, 241-246.

Voyer RA, Hennekey RJ (1972) Effects of dissolved oxygen on two life stages of Mummichog. Progressive Fish-Culturist, **34**, 222-225.

Warren LM (1977) The ecology of *Capitella capitata* in British waters. Journal of the Marine Biological Association of the United Kingdom, **57**, 151-159.

Webb PW, Brett JR (1972) Oxygen consumption of embryos and parents, and oxygen transfer characteristics within the ovary of two species of viviparous seaperch, *Rhacochilus vacca* and *Embiotoca lateralis*. Journal of the Fisheries Research Board of Canada, **29**, 1543-1553.
